# The Complexity of the Holobiont in the Red Sea Coral *Euphyllia paradivisa* under Heat Stress

**DOI:** 10.3390/microorganisms8030372

**Published:** 2020-03-06

**Authors:** Dalit Meron, Keren Maor-Landaw, Gal Eyal, Hila Elifantz, Ehud Banin, Yossi Loya, Oren Levy

**Affiliations:** 1 The Mina and Everard Goodman Faculty of Life Sciences, Bar-Ilan University, Ramat Gan 5290002, Israel; dalitmeron@gmail.com (D.M.); keren.maor@live.com (K.M.-L.); g.eyal@uq.edu.au (G.E.); hila.elifantz@gmail.com (H.E.); ehud.banin@biu.ac.il (E.B.); 2 Morris Kahn Marine Research Station, University of Haifa, Haifa 3498838, Israel; 3 School of BioSciences, University of Melbourne, Melbourne, VIC 3010, Australia; 4 ARC Centre of Excellence for Coral Reef Studies, School of Biological Sciences, The University of Queensland St. Lucia, Qld 4072, Australia; 5 The Institute for Nanotechnology and Advanced Materials, Bar-Ilan University, Ramat Gan 5290002, Israel; 6 Department of Zoology, Tel-Aviv University, Tel Aviv 6997801, Israel; yosiloya@post.tau.ac.il

**Keywords:** holobiont, heat stress, algal symbiont, coral, Euphyllia paradivisa, apo-symbiont, microbial communities

## Abstract

The recognition of the microbiota complexity and their role in the evolution of their host is leading to the popularization of the holobiont concept. However, the coral holobiont (host and its microbiota) is still enigmatic and unclear. Here, we explore the complex relations between different holobiont members of a mesophotic coral *Euphyllia paradivisa*. We subjected two lines of the coral—with photosymbionts, and without photosymbionts (apo-symbiotic)—to increasing temperatures and to antibiotics. The different symbiotic states were characterized using transcriptomics, microbiology and physiology techniques. The bacterial community’s composition is dominated by bacteroidetes, alphaproteobacteria, and gammaproteobacteria, but is dependent upon the symbiont state, colony, temperature treatment, and antibiotic exposure. Overall, the most important parameter determining the response was whether the coral was a symbiont/apo-symbiotic, while the colony and bacterial composition were secondary factors. Enrichment Gene Ontology analysis of coral host’s differentially expressed genes demonstrated the cellular differences between symbiotic and apo-symbiotic samples. Our results demonstrate the significance of each component of the holobiont consortium and imply a coherent link between them, which dramatically impacts the molecular and cellular processes of the coral host, which possibly affect its fitness, particularly under environmental stress.

## 1. Introduction

A holobiont is defined as the combination of all organisms that compose a specific system and combine their adaptations to meet environmental challenges as a consortium. The fitness of the whole consortium to the changing environment will determine the holobiont’s evolutionary trajectories. There are many examples in nature for these complex systems, such as rhizospheres of plants that comprise their roots as well as bacteria and fungi that surround them [1,2], gut and skin biomes [3,4,5] and others. In the last decade, the study of corals has begun to take a holobiont approach, in which the animal host is examined together with its associated diverse microbial community that includes bacteria, archaea, fungi, protists and viruses [6,7]. Despite an appreciation that interactions between corals and associated microbes are likely to have a dramatic influence on coral physiology and health [8], our understanding of the role and the crosstalk between the microbiota and its host is still limited. Many studies have focused on coral–pathogen interactions [9,10], although beneficial symbioses, such as the delivery of photosynthesis products from symbiotic algae [11], resistance to pathogens [12], and nitrogen fixation [13], have also been described.

About 1000 distinct microbial ribo-types have been documented in association with corals, including the representatives of 16 bacterial divisions [14]. This association may be coral specific [6] or even fraction specific, i.e., mucus, tissue, or skeleton [15,16,17], and the relationship with the host is dynamic and complex [18,19,20]. Many studies have described a rapid shift in the microbial population in response to environmental changes such as temperature and bleaching [21] and/or ocean acidification [16,22]. This has led to the hypothesis that the holobiont utilizes changes in its microbial community to adapt to changing environmental conditions more rapidly than possible by mutation and selection [23], allowing for a quicker holobiont evolution.

Recent studies have presented evidence suggesting a new perspective on the cnidarian holobiont, where Symbiodiniaceae–bacterial interactions play a key role in the holobiont nutrient cycling, symbiont stability, and overall fitness (recently reviewed by [24]). The metabolic capabilities of the symbiotic bacteria, and their localization within and around the algal symbionts, support this hypothesis [17]. Facilitation of coral-algae endosymbiosis by bacterial symbionts may be particularly important in mesophotic coral ecosystems (MCEs) whose deeper environment drives niche differentiation, increasing the numbers of microbial symbionts compared to shallow water corals [17,25]. In addition, the microbial assemblage of MCEs differs from that of shallow reef corals [8], reflecting environmental differences in light, temperature, pressure, nutrient availability, and amount of irradiance [26].

The mutualistic symbiosis between corals and their symbiotic micro-algae (Symbiodiniaceae) (formerly *Symbiodinium*) [27] is critical for the success of corals and coral-reef ecosystems [28]. Symbiotic dinoflagellates originated approximately 160 mya [27] and diversified together with their coral hosts during the middle to late Jurassic (~176-161 mya) [29]. Although climate change induced coral–algal symbiosis breakdown and mass coral bleaching events have increased in frequency, intensity, geographical extent, and depth over the past decades [30], MCEs have proved relatively resilient compared to corals in shallow reefs [31,32].

The aim of this study was to investigate the complex relationships between different holobiont members of a mesophilic coral (*Euphyllia paradivisa*) and the contribution of the micro-symbionts to the host’s cellular processes at the transcriptional level alongside examination of the microbial and physiological patterns. We hypothesize that impairment of the holobiont integrity by modifying one or more of its micro-components will critically affect its response during thermal conditions. This study’s goal was to examine the influence and contribution of each of the micro-elements of the holobiont under heat stress. To try and answer this challenging complexity, we choose to work with the coral *E. paradivisa,* since it is one of the most abundant species in the mesophotic coral reefs of Eilat, Israel, exhibiting high physiological plasticity such as tolerance to high irradiance, high competitive abilities, and successful symbiont adaptation [33]. Two lines of corals were utilized in our study; (1) with algal symbionts, (2) without algal symbionts. These lines were subjected to increasing water temperatures and then exposed to antibiotic treatment. All lines were analysed using metagenomic approaches together with bioinformatics tools and molecular, microbiological and physiological techniques.

## 2. Materials and Methods

### 2.1. Coral Sampling and Experimental Design

**Figure 1:**
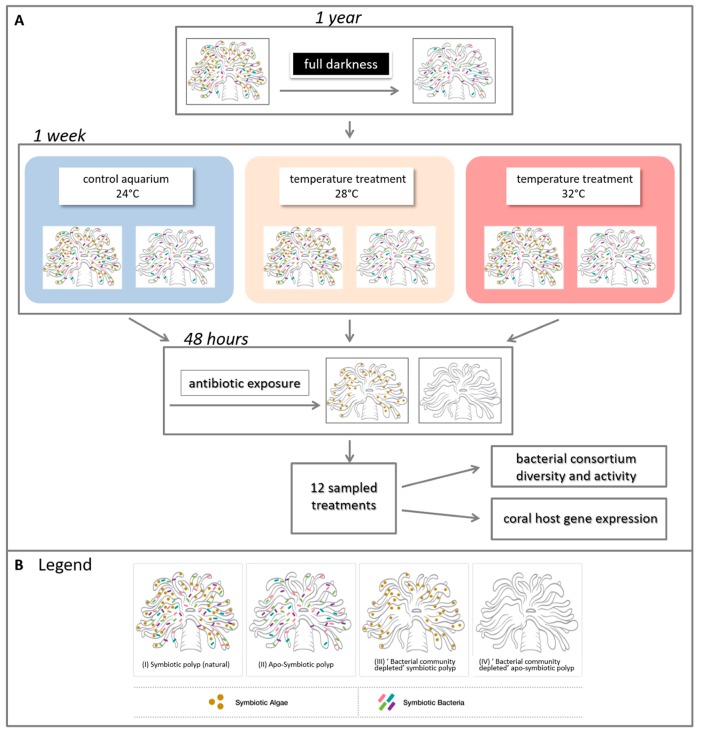
Experimental design. (**A**) Experiment layout and (**B**) legend of four generated symbiotic states. Four adult colonies of *Euphyllia paradivisa* were collected from 40–60 m depth from the Gulf of Eilat and fragmented. Half of the fragments were kept in darkness for a year to generate a full apo-symbiotic type. Symbiotic and apo-symbiotic fragments were subjected to heat-stress for a week, at 28 and 32 °C, and, as a control, 24 °C. After this time, half of the fragments (from each temperature and symbiotic state) were exposed to antibiotics for 48 h to create a “bacterial community depleted” type. In total, 12 treatments (2 symbiotic morphs × 3 temperatures × 2 bacterial states = 12) were analysed for associated microbial communities and host gene expression (see Tables S1 and S2 for additional details).

Four adult colonies (# 14, 15, 17, and 18) of *E. paradivisa* were collected at 40–60 m depth from the coral reefs of Eilat, Israel, fragmented to nubbins of ca. 5 cm and placed in running open-circuit seawater aquaria at the Inter-University Institute (IUI) for Marine Sciences in Eilat, Israel. Colony fluorescence was evaluated in situ through a yellow long-pass filter upon excitation of the corals with a ~450-nm light source (Nightsea). Two types of fluorescence were identified; green and yellow (14, 18 and 15, 17 respectively), as indicated previously [34]. Following a similar layout as we recently conducted [35], for 12 months, half of the fragments were kept under ambient light (symbiotic = S) (see Table S1 in Eyal et al. 2016) while the other half were kept in darkness to generate an apo-symbiotic (lacking the endosymbiotic algae) (apo-symbiotic = AS) state (see Figure 1). The two sets (S and AS) were grown with a running seawater system. This way, the corals were naturally fed in a heterotrophic manner. All the coral fragments remained viable for the whole year, exhibited a performance of fully extended tentacles and were documented with low mortality rates [33]. To evaluate the maximum quantum yield of photosystem II of the algal symbionts (photosynthesis yield *Fv/Fm*), polyps of *E. paradivisa* from all treatments were examined under a PAM (pulse amplitude modulator) fluorometric device (Diving-PAM, WALZ, Germany). The fragments that were kept in ambient light achieved high symbiont photosynthetic efficiency (yearly average: 0.739 (+/− 0.007 95% CI)), while no fluorescence efficiency was detected in the apo-symbiotic fragments.

Following a period of 12 months, each set was divided into three close-circuit aquarium systems with artificial seawater (Brightwell Aquatics, PA, USA) at an ambient temperature of 24 °C at Bar-Ilan University, Israel. All fragments were kept under a light intensity of 15–52 µmol photons m^−2^ s^−1^ during day time, which is characteristic of their natural habitat conditions. After a week of acclimation, the aquaria temperatures were gradually increased from 24 to 32 °C (~1 °C per day), resulting in two sets of treatments (symbiotic and apo-symbiotic) at three temperatures: 24, 28 and 32 °C (Figure 1A). The corals were fed twice with freshly hatched Artemia *sp. nauplii* during this week of acclimation.

Following a week of incubation with the respective temperature, a short incubation with antibiotics was performed on half of the fragments, in order to evaluate the relationship between the coral and its associated bacteria under heat stress. Each fragment was placed in a 1 L sterile beaker containing filtered seawater (FSW) (the beakers were kept under the same original temperatures (24, 28 and 32 °C). The antibiotic combination included 0.1 mg/mL of nalidixic acid, ampicillin and streptomycin for 48 h according to [36,37] with minor modifications of concentrations (based on preliminary experiments). Following the antibiotic exposure, the fragments were transferred to clean FSW for 24 h of recovery, and then sampled for various analyses (see below). Thus, finally, the experiment included samples of four sets of coral morphs (Figure 1): (i) Symbiotic polyp, including its natural microbiota (bacteria and endosymbiotic algae); (ii) Apo-symbiotic polyp (iii) “bacterial community depleted” symbiotic polyp (iv) “bacterial community depleted” apo-symbiotic polyp, at three temperatures, resulting with 12 treatments in total (Figure 1B). The samples for all analyses were snap frozen in liquid nitrogen and kept at −80 °C until further analysis.

### 2.2. Bacterial DNA Extraction and NGS Analyses

DNA for next-generation sequencing (NGS) of bacterial diversity was extracted from the homogenized coral samples (tissue and skeleton) (Table S1) using the UltraClean Soil DNA kit according to the manufacture’s guidelines (MoBio, Carlsbad, CA, USA). Bacterial 16S rRNA genes were amplified using 27F and 1100R primers and then a nested PCR was performed using barcode 27F primer and 515R primer [38,39]. The tagged amplicons NGS was performed on an IonTorrent (Applied Biosystems, Waltham, MA, USA) at the Bar-Ilan University sequencing unit for 64 coral samples (see Table S1) (total 289,579 sequences). Sequences were prepared for analysis (trimmed, aligned, screened) using MOTHUR v.1.32.1 [40] as detailed below. First, the sequences were trimmed using the following settings: ambiguities; 0, maximum homopolymers allowed; 7, quality window average threshold; 20, quality window size; 25, and one and two mismatches were allowed for the barcode and the primers, respectively. After trimming, 430,800 sequences were available for further analysis and the average sequences per sample was 6500. The sequences were further aligned against the silva.seed_v119 database and screened according to the median values of ‘start’ and ‘end’ positions of the alignment. Preclustering was used to group sequences with difference values set to 2 and the clusters were checked for chimeras with the uchime [41] module in MOTHUR. Sequentially, a total of 6990 chimeras were found and removed from the dataset. Operational Taxonomic Units (OTUs) were determined by a distance matrix with a cut-off of 0.15 and clustering, while rare OTUs (1–2 sequences) were removed. The taxonomy of the OTU was determined by comparing the sequences to the silva bacteria database. Uncertainties were checked in the blast algorithm in NCBI and corrected accordingly. Chloroplast sequences were removed from the dataset. Statistical analysis was done in PAST statistical analysis software [42]. The sequences in this study were deposited in the GenBank nucleotide sequence database under study accession SRP133996.

### 2.3. Coral RNA Extraction and Sequencing

The total RNA was extracted from each of the fragments (tissue and skeleton) from the different treatments using TRIzol reagent (Invitrogen Life Technologies, Carlsbad, CA, USA) according to the methods described previously [43]. To assess RNA quality (RIN > 8.5), RNA samples were analysed using a NanoDrop 1000 spectrophotometer (ThermoScientific, Wilmington, DE, USA) and 2100 Bioanalyzer (Agilent, Waltham, MA, USA).

1.5 µg RNA from each sample (*n* = 33, including only the samples with the highest quality extracts) was prepared using the Illumina TruSeq RNA Library Preparation Kit v2, according to the manufacturer’s protocol. Using the multiplexing strategy of the TruSeq protocol, 33 libraries of the four sets (symbiotic, apo-symbiotic, “Modified bacterial community” symbiotic and apo-symbiotic polyps) ran on three lanes of an Illumina HiSeq2000 machine. Each sample obtained an average of ~10 million paired-end reads. The sequencing was conducted by the NGS unit of the Technion research and development foundation LTD (http://isu.thecnion.ac.il), Israel. The Fastq files have been deposited in an SRA database under the BioProject ID: PRJNA385711.

### 2.4. Coral Transcriptome Assembly and Annotations

RNA-seq reads were searched against three target databases: NCBI NR (non-redundant protein database of all life domains) using Diamond, the Symbiodiniaceae database (http://zoox.reefgenomics.org) using BLASTN, and against the *Fugacium* kawagutii (formerly *Symbiodinium* kawagutii) [27] unigene database using BLASTN. The reads mapped to bacteria or plants, but not to any animal sources, were filtered out. The remaining reads were further used for transcriptome assembly using Trinity (version 2.2.0) [44]. We used the program RSem [45] to map non-contaminated reads to the transcriptome assembly, and then read counts per gene were normalized as CPM (Count Per Million) using the TMM method, in EdgeR (within R 3.1.2) (https://bioconductor.org/packages/release/bioc/html/edgeR.html). In total, 31,664 Trinity genes with CPM > 5 were found, and among them 17,892 matched animal sequences. This identification was done by mapping the contigs to several databases: NCBI NT database using BLASTN, and the *Nematostella* protein database using BLASTX. In addition, a reciprocal Blast search was conducted against the human Ensembl transcripts, using CRB-Blast [46].

### 2.5. Differential Expression Analysis and Clustering

Read count normalization was conducted using the program EdgeR [47]. Read counts, for 17,892 non-contaminated putative genes, were normalized using the TMM methods, as count per million mapped reads (CPM), and fragments per kilobase of mapped exon, per million mapped reads (FPKM). Samples were further clustered using multi-dimensional scaling (MDS). Four samples were found to be clustering-outliers and were excluded from the analysis based on their low read counts, quality or putative contamination (see Table S2). DE analysis was conducted using EdgeR GLM method [47], which allows modelling the additive effect of different factors on the total normalized counts, namely symbiont state, temperature, and antibiotic exposure, on the total normalized counts. We included the four factors—colony origin, symbiont state, temperature, and antibiotic exposure—in the GLM model. We first identified per-gene cases of significant symbiont effect (adjusted *p* value < 0.05), significant temperature change effects (32 vs. 24 °C, 28 vs. 24 °C, and 32 vs. 28 °C). A significant factor effect (adjusted *p* value < 0.05) was defined as greater than a two-fold change. Similarly, we searched for genes with significant effect of antibiotics treatment.

For heat map representations, relative transcript abundance values were calculated. First, all DE samples were divided into groups, so that each group includes individuals from the same colony, under the same symbiont state. Then, the relative transcript abundance was calculated by dividing every individual FPKM belonging to a specific group (specific colony and symbiont combination), from the mean FPKM of all individuals of the same group. In addition, with the purpose of analysing host gene expression for the different treatments (alongside the combination of factors), we calculated fold changes for each treatment using the relevant control (each heat-stress sample to its 24 °C control while keeping the factors of symbiont state, antibiotic exposure and colony origin constant). An arbitrary cut-off of at least two-fold was chosen to define a differentially expressed gene (DEG). Thus, we generated 8 lists of genes: symbiotic at 28 °C, apo-symbiotic at 28 °C, symbiotic at 32 °C, and apo-symbiotic at 32 °C that their fold change decrease and increase followed the antibiotic exposure.

Functional gene analysis and KEGG pathways analysis was done by David Bioinformatics Resources 6.7 [48,49], using the Homo sapiens orthologs annotations. The GO terms assigned to the contig sequences were exported to the web-based CateGOrizer [50] in order to generate GO slim terms and to count the ancestors’ terms as a percentage of the total GOs.

## 3. Results

In this study, the importance of the symbiont algae and the bacterial community to the physiology of the coral *E. paradivisa* during a heat stress was evaluated. The symbiosis effect was tested by creating apo-symbiotic corals as described above. Pairwise comparisons of the photosynthetic maximal quantum yield (Fv/Fm) of the polyps with algal symbionts showed no significant differences at any temperature, time point or antibiotic exposure (Two-way ANOVA, F interaction = 1.4, *p* = 0.118) and the average Fv/Fm value was 0.6. Apo-symbiotic polyps consistently gave zero yield during the experiment (Figure S1), and, as we showed in an accompanied paper [35] no algal cells and chlorophyll a were detected, as expected. While the presence or absence of symbiosis is relatively straight forward, the bacterial community did not disappear completely due to the antibiotic treatment, but rather the species composition was depleted and shifted, as will be presented and discussed below (see Figure S2 for effect of antibiotic exposure on culturable bacteria abundance).

### 3.1. Clusters in Host Gene Expression and Bacterial Communities

**Figure 2:**
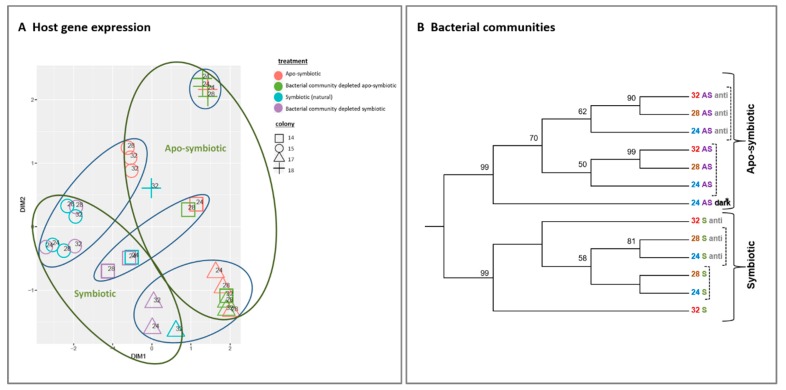
Host gene expression and bacterial communities clustering by symbiotic and apo-symbiotic *E. paradivisa* samples (12 treatments). (**A**) Multi-dimensional scaling (MDS) clustering of 29 tested samples (15 symbiotic and 14 apo-symbiotic; see Table S2). Colony origin is indicated by a distinctive shape, the preliminary treatment by a colour (see legend), and the temperature is indicated within the figure. Clusters formed according to apo/symbiotic state are marked with green circles, and clusters formed according to colony origin are marked with blue circles. (**B**) Cluster analysis of microbial community 16S rRNA NGS libraries (*n* = 289,579) generated from 13 *E. paradivisa* samples (12 treatment including one group “24 AS dark*” as control, see below and Table S1)). An unrooted neighbour-joining tree of communities by 16S rRNA gene NGS. Bootstrap values represent 100 iterations. Sequences retrieved were analysed using MOTHUR software [40]. The curly brackets indicate the main cluster segregation by symbiotic and apo-symbiotic types, while the dashed brackets indicate an inner subdivision by antibiotic treatment. The sample “24 AS dark*” represents apo-symbiotic polyps that were kept in the dark as control. Labels are coloured according to temperature treatment and symbiotic state type.

We inspected the response of host gene expression and its bacterial community composition to the increasing temperature and antibiotic treated (Figure 2). The multi-dimensional scaling (MDS) clustering displays the gene expression variance of the 29 tested samples. The samples were grouped into clusters primarily by symbiotic morph factor (symbiotic vs. apo-symbiotic) and colony origin (original coral) without apparent effect of temperature or antibiotic treatment (Figure 2A).

Similarly, the bacterial communities clustered according to the coral state first (symbiotic or apo-symbiotic) but in contrast to gene expression, we see here subdivision according to antibiotic exposure rather than temperature (dashed line in Figure 2B). Another observation to note is that in the symbiotic corals, the 24 and 28 °C bacterial communities clustered together and separately from the 32 °C treatment. But, in the apo-symbiotic corals, the 28 °C and 32 °C treatments clustered together and separately from the 24 °C treatment. Also worth noting is that the separation between the antibiotics treated and un-treated groups was more distinctive in the apo-symbiotic polyps (Figure 2B).

### 3.2. The Diversity of Coral Host Associated Bacteria

**Figure 3:**
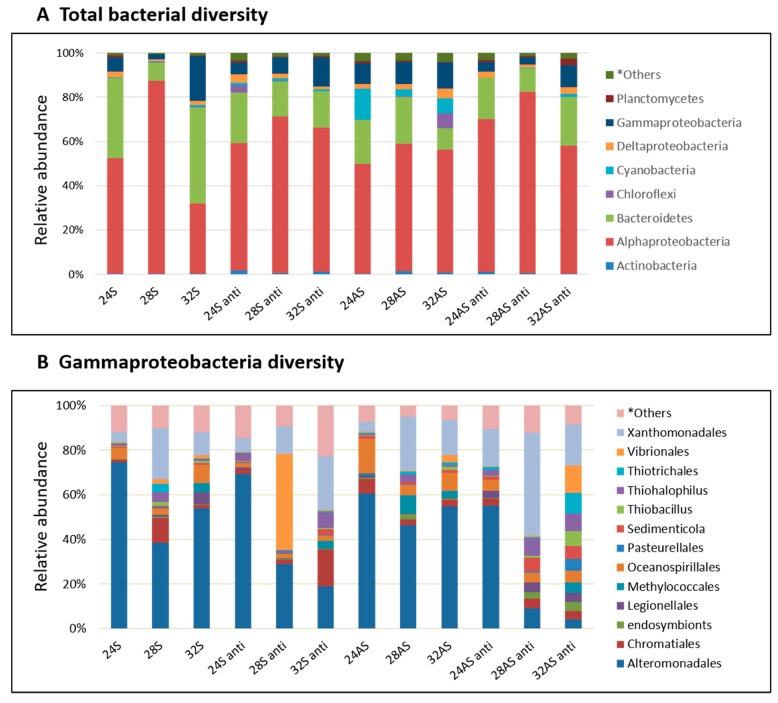
Bacterial composition and relative abundance of symbiotic and apo-symbiotic *E. paradivisa* samples (12 treatments). (**A**) total bacterial diversity divided by phylum and (**B**) Gammaproteobacteria class diversity divided by order. *Others includes all groups under 2%.

We further looked at the variations between the bacterial communities in the different treatments by inspecting the bacterial community composition (Figure 3A). The communities in the treated corals were compared to the community of the symbiotic corals kept in 24°C (baseline community), as these are the corals that simulate most closely the current conditions in the Gulf of Eilat. The baseline community was dominated by Bacteroidetes (45%), alphaproteobacteria (30%) and gammaproteobacteria (20%). In all treatments, the abundance of Bacteroidetes and gammaproteobacteria decreased compared to the baseline community, while the abundance of alphaproteobacteria increased. An interesting observation was the increase in the abundance of cyanobacteria in the apo-symbiotic corals in all three temperatures. The relative abundance of cyanobacteria in apo-symbiotic corals reduced to less than 1% after antibiotic exposure. (Figure 3A). The Shannon index (measures species diversity based on species richness and evenness) was highly variable between the biological replicates and treatments (ranging between 0.4 and 5.2).

As stated above, alphaproteobacteria increased in abundance in all conditions compared to the baseline community. Sphingomonadaceae was the most abundant family (average of about 83% of alphaproteobacteria) and the dominant OTU genus *Sphingomonas* was identified by NCBI database comparison as *Sphingomonas paucimobilis* (GenBank accession number KX055885, 98% identity). An increase was observed also in another group, the Rhizobiales, including diazotrophs members, in the apo-symbiotic polyps (5 and 5.5% of alphaproteobacteria) at 28 and 32 °C, respectively. Meanwhile, in the symbiotic polyps, lower percentages were observed (1.8 and 2.46% of alphaproteobacteria) at 28 and 32 °C, respectively.

While gammaproteobacteria made only 20% of the microbial community in the 24 °C symbiotic colonies, it is an important member of the holobiont, as some of the pathogens known for coral bleaching and other diseases are associated with that family [51,52] Therefore, a more careful inspection was done on the specific class of gammaproteobacteria in the different conditions (Figure 3B). The most striking observation was the difference between the community compositions of the control and colonies that were exposed to the most extreme treatment–the apo-symbiotic colonies treated with antibiotics at 28 and 32 °C. While the control colonies were dominated by Alteromonadales, the 28 and 32 °C treated colonies were more diverse and included 7–9 families with equal percentages and one dominant family in the 28 °C colonies and three co-dominants in the 32 °C colonies. One family that increased its abundance in those and other colonies was the Xanthomonadales. Other families that showed an increase in some of the colonies included Chromatiales, Oceanospirillales, and Vibrionales, all of which on the expense of Alteromonadales.

**Figure 4:**
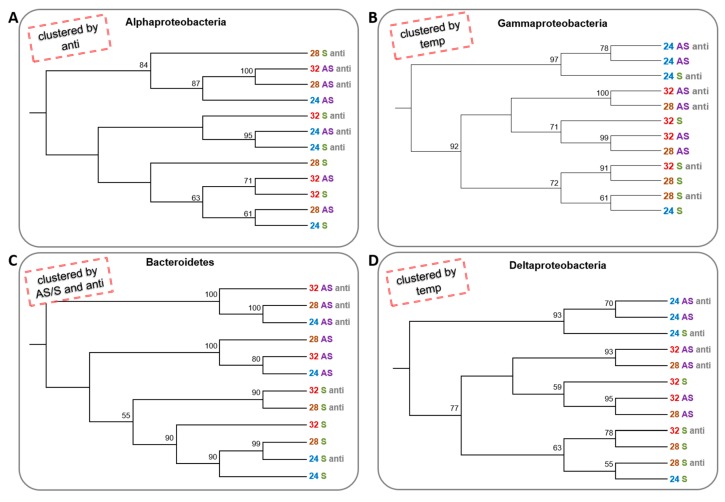
Cluster analysis of microbial community from four bacterial groups of 16S rRNA NGS libraries generated from *E. paradivisa* samples. (**A**) Alphaproteobacteria (**B**) Gammaproteobacteria (**C**) Bacteroidetes and (**D**) Deltaproteobacteria. An unrooted neighbour-joining tree of communities by 16S rRNA gene NGS. Bootstrap values represent 100 iterations. Retrieved sequences were analysed using MOTHUR software [40]. The headlines (in the red dashed box) indicate the main factor or factors that affect the clustering pattern. Labels are coloured according to the temperature treatment and the symbiotic state.

To determine whether the change in conditions caused a change in the species composition of the dominant groups (alpha-, delta- and gammaproteobacteria) and phylum (Bacteroidetes), a neighbour-joining test was performed for each separately. The alphaproteobacteria clustered into three clusters; one that contained only communities that were not treated with antibiotics, regardless of the temperature and the symbiotic state, and two clusters that contained communities that were treated with the antibiotics. However, there was almost no importance to the temperature or the symbiotic state of the corals (Figure 4A). In contrast, gammaproteobacteria and deltaproteobacteria were clustered primarily by temperature, where three of the 24 °C treatments were in one cluster and the 28 and 32 °C colonies composed another branch, which had two main clusters. One cluster contained only symbiotic corals and the other apo-symbiotic corals. The antibiotics treatment did not have any effect on the gammaproteobacteria (Figure 4B,D). Bacteroidetes had the most “organized” clustering, where the apo-symbiotic treated with antibiotics communities made one cluster, the apo-symbiotic with no antibiotics made a second cluster, and the symbiotic communities made a third cluster, with some separation between the antibiotics treated and untreated communities. However, temperature change did not have any effect on Bacteroidetes clustering. In fact, the Bacteroidetes tree most resembled the tree cluster of the whole bacterial community (Figure 4C and Figure 2B, respectively).

### 3.3. Gene Expression Analysis of the Coral Host

One aspect that was examined in this study was the response of the coral to the changing environment (temperature, symbionts presence, microbial community changes) by transcriptome analysis. The symbiont state had the most effect on gene expression, whereas all antibiotic-treated versus all non-treated samples yielded the lowest number of DEGs (Figure S3). The effect of temperature was most evident between 32 and 24 °C, with 1300 DEGs detected. The first temperature increase, at 28 °C, yielded more DEGs than the transition between 28 and 32 °C. Moreover, most (72.5%) of the DEGs were down-regulated from 24 to 28 °C, but were mostly up-regulated (72%) after a further increase to 32 °C. Overall, across the range of 24 to 32 °C, the percentages of up-regulated versus down-regulated genes are similar.

**Figure 5:**
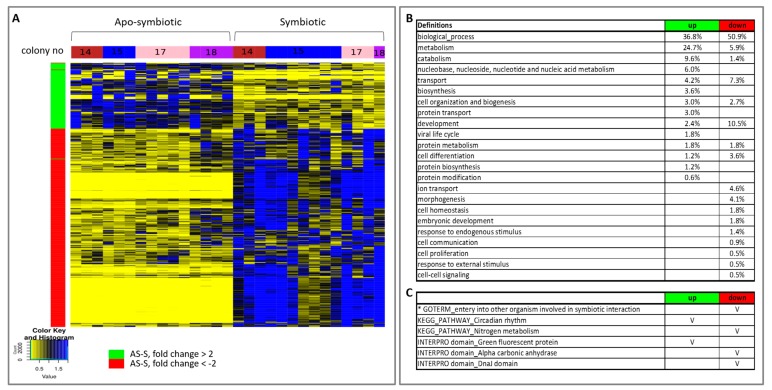
Heat map of hierarchical clustering and enrichment analysis of all apo-symbiotic vs all symbiotic samples. (**A**) A heat map showing the relative transcript abundance levels of differentially expressed genes (*p* value < 0.05) having a mean fold change of > 2 or < −2, between apo-symbiotic samples and symbiotic samples. The tested groups, colonies 14, 15, 17 and 18, are marked in the horizontal colour bar: red, blue, pink and purple, respectively. (**B**) Enriched Gene Ontologies (GOs) in the up-regulated and down-regulated groups of apo-symbiotic vs symbiotic analysis. GO slim terms, generated by the web-based tool CateGOrizer [50], are presented as a percentage of the total GOs of the group. (**C**) Other GO, KEGG pathways, INTERPRO domains of interest that were significantly enriched in the up- or down-regulated groups are marked with V. An asterisk marks a result from a reciprocal BLAST analysis.

The heat map of the hierarchical clustering of gene expression of all *E. paradivisa* samples further highlights the clustering of the samples according to the symbiotic state (Figure 5A). The relative transcript abundance levels of significant differentially expressed genes, between all apo-symbiotic samples and all symbiotic samples, are illustrated. Enrichment Gene Ontology (GO) analysis of the up- and down-regulated genes revealed the major cellular differences leading to separate clustering of the apo-symbiotic and symbiotic *E. paradivisa* samples (Figure 5B,C). Higher percentages of metabolism, catabolism, and biosynthesis processes are more up-regulated in the apo-symbiotic samples, while developmental processes, morphogenesis, embryonic development and cell proliferation are more down-regulated in the apo-symbiotic corals. In addition, transport and ion transport genes seem to be more down-regulated in the apo-symbionts. Other interesting enrichment analyses in KEGG pathways and INTERPRO domains are presented in Figure 5C. Green Fluorescent Protein (GFP) was found in the up-regulated group of apo-symbiotic vs. symbiotic *E. paradivisa.* Figure S4 further shows that the expression values of a contig possessing this domain is significantly higher in the apo-symbiotic state than in the symbiotic type.

**Figure 6:**
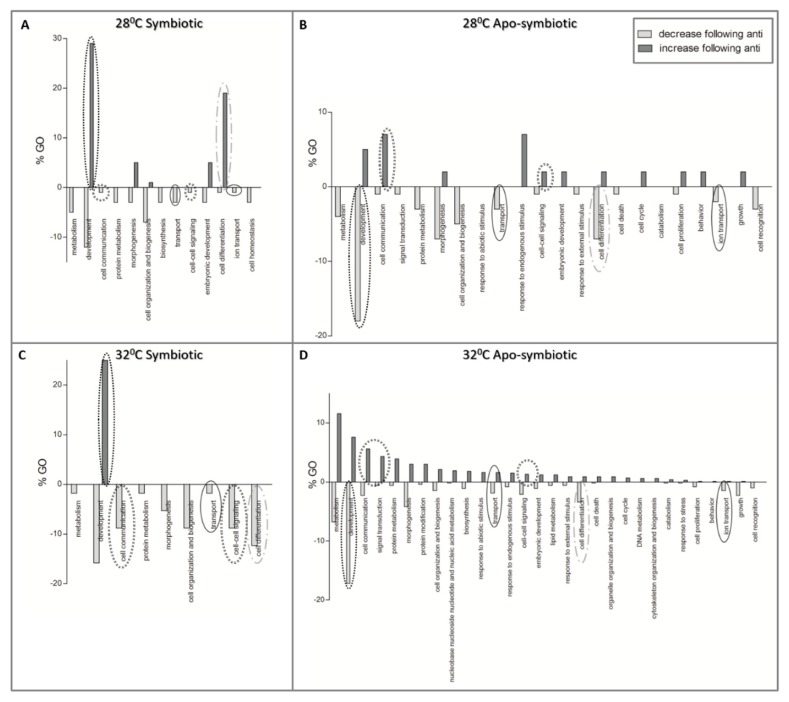
The effect of antibiotic exposure on coral host gene expression. Enriched Gene Ontologies (GOs) of differentially expressed genes which their fold change was either lower (light grey) or higher (dark grey) following the antibiotic exposure. GO slim terms, generated by the web-based tool CateGOrizer [50], are presented as a percentage of the total GOs. (**A**) symbiotic *E. paradivisa* at 28 °C, (**B**) apo-symbiotic *E. paradivisa* at 28 °C, (**C**) symbiotic *E. paradivisa* at 32 °C, and (**D**) apo-symbiotic *E. paradivisa* at 32 °C. Several GOs of interest are marked with circles: development, communication, differentiation, and transport in the cell.

Figure 6 presents the results of the enrichment analysis as GOs (percentages of slim terms) affected by antibiotic exposure. More cellular processes were more affected by antibiotics in the apo-symbiotic samples (Figure 6B,D), than in the symbiotic ones (Figure 6A,C). Moreover, in the symbiotic samples there are more processes that decrease following antibiotic exposure than increased processes. GO of development is more triggered by community change in symbiotic samples while it is more suppressed following the community change in apo-symbiotic samples, regardless of the temperature. In contrast, the GO of cell communication is reduced in symbiotic but increased in apo-symbiotic samples. 

## 4. Discussion

Today, the holobiont concept is being emphasized in studies of different ecosystems to understand the potential influence of global change on host biology [21,53,54,55]. In particular, it is important to look at all the components of the holobiont in the case of symbiosis, as these symbionts may be crucial for the fitness of the holobiont and its evolution in a changing environment. For the purpose of this experiment, we artificially generated a coral morph lacking its symbiotic algae, and a morph with altered natural prokaryote microbiota, that, according to our knowledge, cannot be found in the natural environment. These experimental manipulations firstly provide a proof-of-concept that at least one coral species can be viable as apo-symbiotic and thus suitable for such studies in the future. Moreover, this enabled us to explore the interconnections between the holobiont components by examining the different morphs.

The four *E. paradivisa* colonies from the mesophotic reef used in this study were selected to provide an appropriate representation of possible biological and genetic variability. Our results clearly demonstrate that the colony has a strong influence on coral gene expression pattern (Figure 2A). This outcome corresponds with previous documentation illustrating that gene expression varies in coral individuals [56,57] according to colony size, shape and thermal/light life histories [58,59]. Thus, future coral holobiont studies should consider the difference between the colonies and the variability between genotypes within the same species. Although in our study, the colony was not a significant parameter determining the clustering of bacterial communities, bacteria species diversity did vary between colonies.

In the current study, the presence of algal symbionts was the key factor influencing other components of the coral holobiont, outranking bacteria, temperature, or colony origin, as we discovered post sampling. However, we note that the duration of the antibiotic exposure was potentially insufficient for triggering a more substantial effect. This finding corresponds with our work showing the algal symbiont effect on coral cellular processes under stress [35]. There were two distinct clusters representing all symbiotic vs. apo-symbiotic host’s RNA samples (Figure 2A) that are further highlighted by a hierarchical clustering heat map (Figure 5A). A possible explanation for this is that apo-symbiotic polyps, which are missing the autotrophic component of their holobiont, may need to increase their own levels of metabolism, catabolism, and biosynthesis to compensate [60] for the lack of photosynthetically fixed carbon that is translocated regularly to the host in symbiotic corals [28]. Therefore, the observed increase in catabolism could be a manifestation of the elevated energetic demands of a cell lacking the algae supplier. Alternatively, as inputs of assimilated photosynthates decrease, the apo-symbiotic coral may shift to a more heterotrophy-based diet [61], which manifests in an increase in catabolism processes [62].

Similarly, the reduction in transport processes observed in the apo-symbiont fragments could reflect the lack of the intimate molecular communication and mutual transport exchange of resources that usually take place between coral and algal symbiont [63,64]. The down-regulation of carbonic anhydrase (CA) in the apo-symbiotic polyps supports previous reports that CA activity and transcript amounts are higher in symbiotic cnidarians than in apo-symbiotic cnidarians [65,66] and can be induced in the presence of algae [67]. CA catalyses the hydration of CO_2_ to HCO_3_^−^ and is thought to play a significant role in the calcium carbonate assimilation of scleractinian corals [68,69].

A possible explanation for the decrease in GOs related to cell development and morphological changes in the apo-symbiotic samples could be related to the differentiation of phagosomes to form the symbiosome (a distinct cytoplasmic vacuole containing the symbiotic alga) [70,71]. A GO found in the down-regulation group may indicate a direct relationship to symbiotic interaction (“entry into other organism involved in symbiotic interaction”) (Figure 5C) and highlights the cellular differences between apo-symbiotic and symbiotic polyps. An additional reason for the regulation of genes could be that the chloroplasts of photosynthetic symbionts represent a prime source of ROS [72], and possible oxidative stress [72,73] that could stimulate the expression of chaperones as a protective response. Supporting this theory, the DnaJ (hsp40) domain was decreased in the apo-symbiotic polyps (Figure 5C).

The increase in GFP transcripts in the symbiont-free polyps (Figure 5C and Figure S4) could represent a host photo-protective response for regulating the light environment in the absence of the photosymbionts, as has been documented in bleaching events [74,75,76]. Further, this could be the result of an unaccustomed sunlight and UV exposure encountered by polyps transferred to regular aquaria after a year in the darkness. The greatest increase in GFP was seen in samples from the yellow fluorescence type in colony 17, which exhibited two fluorescence emission peaks: green and yellow [34]. We have previously shown that the *E. paradivisa* yellow type loses the yellow peak in the dark, while the green fluorescence remained unaltered [34]. These gene expression findings highlight the profound nature of the intimate relations between the coral host and the algal symbiont, ranging over molecular pathways, from metabolism, calcification, and transport to morphological changes.

In addition to the symbiont presence, temperature also had an effect on coral gene expression. The treatment of 32 °C triggers large numbers of DEGs (Figure S3). This pattern of gene expression could reflect a response to cellular shock/trauma, where the down regulation of *E. paradivisa* genes is mitigated as the stress continues and eventually transitions into the up-regulation of genes. It is noteworthy that no visual physiological changes (no bleaching, alterations in tissue integrity, expanded tentacles) were documented throughout the heat-stress experiment. Photosynthetic maximal quantum yield was not affected by the temperature treatment nor antibiotic exposure, which might indicate that the intensity or duration of these might not have been sufficient for triggering photo-physiological changes in *E. paradivisa*.

The observed changes in coral gene expression following antibiotic exposure imply the presence of a profound link between the host and its associated bacteria. Moreover, our results demonstrate that antibiotic exposure changed more cellular pathways in polyps that lacked algal symbionts (Figure 6B,D), indicating that Symbiodiniaceae may mitigate the magnitude of the antibiotic effect on the coral. However, temperature treatment and antibiotic exposure did not cause bleaching and tissue loss as described by Gilbert et al. (2012) [37]. The differences between the studies may be due to different antibiotic exposure time, the delta temperature, or the studied species.

The importance of the algae to the other members of the holobiont has been demonstrated previously by Bourne et al. (2013), who showed that the photosynthetic algal symbionts determine the composition of marine invertebrate-associated bacteria [77]. Littman et al. (2009, 2010) also reported an effect of Symbiodiniaceae on coral-associated bacterial communities of juvenile corals, but only when the holobiont was exposed to a short-term heat stress [55,78]. Changes in the composition of Symbiodiniaceae endosymbionts and in the photosynthetic products released into coral tissues can affect the composition of coral mucus and thus indirectly impact the associated bacteria [78,79]. The causative pathogen *Vibrio shiloi* was found to infect only mucus-containing algal symbionts [80]. Compounds such as DMSP, produced by the algal symbionts, can influence the microbial communities by providing nutrient sources available for their metabolism [77,79]. More than 65% of the bacterial genera known to utilize DMSP compounds have also been reported to be associated with corals [81]. A further example of Symbiodiniaceae–bacteria interactions relates to the association of coral bleaching with changes in the coral microbiota [82].

The strong clustering of the bacterial communities associated with symbiotic or apo-symbiotic fragments (Figure 2B) indicates that the algal symbiont also has a dramatic impact on the associated bacteria. A control sample kept in the dark, clustered within the apo-symbiotic cluster, supporting the supposition that the symbiotic state, has a major influence on bacterial composition. This influence is mostly expressed in the bacterial taxa Bacteroidetes, one of the dominant groups (Figure 4C).

The bacterial communities were dramatically affected by antibiotic treatment and the symbiont state but could, themselves, also affect the holobiont. Three dominant bacterial groups were found in all samples, including Alphaproteobacteria and Gammaproteobacteria, previously described to be the most dominant classes of bacteria associated with reef invertebrates [77] and in particular with corals [8,26,83]. Previous studies reported that increases in temperature resulted in changes of bacterial community composition, including known coral pathogens [12,21,73,84,85]; however, they may be associated with the coral’s variable temperature regime [53]. Sphingomonas (Alphaproteobacteria), which was the most abundant sequence in our samples, have been previously linked to coral diseases [86,87]. The Vibrionaceae family (Gammaproteobacteria), found in 28S anti and 32AS anti (Figure 3B), has been previously linked with heat stress and coral bleaching [10,21,51,82], even though no signs of disease or coral bleaching were detected in our study.

Interestingly, the clustering of bacterial communities differed as we examined different groups of bacterial taxa (Figure 3B and Figure 4). The outputted clusters were influenced by the parameters of antibiotic exposure, temperature and symbiotic state. Our results correspond with Vega-Thurber et al. (2009) who demonstrated a differential response of bacterial taxa to various factors [21]. These results emphasize that the response of the associated bacterial communities is complex and comprised of several different ‘layers’ that might behave differently when challenged with various abiotic factors. The Bacteroidetes decrease in the absence of symbiotic algae could be explained by a possible decrease in complex compounds that are favourable for this group [88,89]. Similarly, the decrease in gammaproteobacteria could also be explained by the reduced flux of exudates from the algae [90,91]. In contrast, alphaproteobacteria, which have a better adaptation to a lack of organic compounds and is more susceptible to oligotrophic conditions, could take advantage and increase its abundance [92].

The amounts of Cyanobacteria were significantly increased in apo-symbiotic samples that were not treated with antibiotics. The significance of symbiotic Cyanobacteria to their host was previously demonstrated in sponges [93,94,95] and corals [96,97,98,99]. The Cyanobacteria–sponge relationship was compared to that of corals and their symbiotic micro-algae [100], since Cyanobacteria provide the host with energy by photosynthate translocation and also synthesize a ‘sunscreen’ that enables the holobiont to thrive in a wide range of environments [101]. The increase in Cyanobacteria in our results may indicate an alternative source of photoassimilates [102] and contribute to nitrogen fixation [96,97,98,99,103] for a coral lacking photosynthate-providing algae. Interestingly, Lesser et al. (2004) observed an increase in colonies containing Cyanobacteria with depth, suggesting that Cyanobacteria could sustain Symbiodiniaceae nutrition in low-light environments [99].

Since coral and algae both benefit from nitrogen recycling [104], the decrease in host’s nitrogen metabolism noted in apo-symbiotic polyps could reflect an influence of the symbiotic state too. Higher levels of nitrogen metabolism and transport genes in symbiotic compared to apo-symbiotic *Aiptasia* were previously documented in a proteomic study [62]. These modifications in nitrogen homeostasis in the apo-symbiotic coral may trigger stronger demands for nitrogen, thus enhancing the association with Cyanobacteria for beneficial nitrogen fixation. An increase in coral-associated microbial nitrogen fixation was previously documented in bleached coral, where symbiosis breakdown was induced by monosaccharides supplement [105]. The phenomenon described here highlights an intriguing example of a symbiotic micro-algae—coral—bacteria interface that may facilitate a return to homeostasis following changes in availability of metabolic requirements.

## 5. Conclusions

This study is novel in dissecting how can environmental changes such as short-term heat stress affect the whole holobiont and its components; we characterize the response of three components of the coral holobiont—the coral host, the photosynthetic algae, and the associated bacteria—and show how each of them may influence each other. The originality of our work lies with our experimental approach of generating four distinguished colonies with different symbiotic states, enabling us to try and examine the role of each component separately in the coral holobiont complexity, which may provide evidence about its contribution to the holobiont acclimatization and survival. These data reveal a coherent link between the algae and bacteria-associated symbionts, with important implications for the molecular and cellular processes of the coral. Our findings pose important open questions regarding the specific triggered/deactivated pathways occurring in the coral cells and the possible interactions with the elements of its microbiome. The recently emerged hologenome (the sum of the genetic information of the host and its microbiota) theory argues that some microorganisms may be co-transmitted between generations and thus further supports the key role of the microbial symbiont in the holobiont fitness, adaptation, and evolution [11,106,107]. However, issues concerning the functional importance of the host’s microsymbiont communities and their impact on the holobiont environmental adaptability under stress, and therefore holobiont evolution, remain still partially enigmatic. Further research is crucial for a better understanding of the coral holobiont and the crosstalks of its components, especially in an era of global change.

## Supplementary Materials

Supplementary materials can be found at https://www.mdpi.com/2076-2607/8/3/372/s1.
